# RETRACTION: Optimising Wound Care for Patients with Cirrhosis: A Study of the Effect of Combination Therapy on Wound Healing

**DOI:** 10.1111/iwj.70533

**Published:** 2025-04-18

## Abstract

**RETRACTION:** LinX. , SuJ. , and YangZ. , “Optimising Wound Care for Patients with Cirrhosis: A Study of the Effect of Combination Therapy on Wound Healing,” International Wound Journal 21, no. 2 (2024): e14727, 10.1111/iwj.14727.38356305 PMC10867491

The above article, published online on 14 February 2024, in Wiley Online Library (http://onlinelibrary.wiley.com/), has been retracted by agreement between the journal Editor in Chief, Professor Keith Harding; and John Wiley & Sons Ltd. Following an investigation by the publisher, all parties have concluded that this article was accepted solely on the basis of a compromised peer review process. In addition, the investigation found that the authors provided incomplete ethical approval information for the study. The editors have therefore decided to retract the article. The authors did not respond to our notice regarding the retraction.

**RETRACTION:** X. Lin , J. Su , and Z. Yang , “Optimising Wound Care for Patients with Cirrhosis: A Study of the Effect of Combination Therapy on Wound Healing,” International Wound Journal 21, no. 2 (2024): e14727, 10.1111/iwj.14727.38356305 PMC10867491

The above article, published online on 14 February 2024, in Wiley Online Library (http://onlinelibrary.wiley.com/), has been retracted by agreement between the journal Editor in Chief, Professor Keith Harding; and John Wiley & Sons Ltd. Following an investigation by the publisher, all parties have concluded that this article was accepted solely on the basis of a compromised peer review process. In addition, the investigation found that the authors provided incomplete ethical approval information for the study. The editors have therefore decided to retract the article. The authors did not respond to our notice regarding the retraction.

